# Dendritic mRNAs encode diversified functionalities in hippocampal pyramidal neurons

**DOI:** 10.1186/1471-2202-7-17

**Published:** 2006-02-17

**Authors:** Jun Zhong, Theresa Zhang, Lisa M Bloch

**Affiliations:** 1 Department for Physiology and Pharmacology, State University of New York Health Science Center at Brooklyn, 450 Clarkson Avenue, Box 29, Brooklyn, NY 11203, USA; 2 Cold Spring Harbor Laboratory, One Bungtown Road, Cold Spring Harbor, NY 11724, USA; 3 Agilent Technologies, Integrated Biology Systems, 1 Cragwood Road, South Plainfield, NJ 07080, USA

## Abstract

**Background:**

Targeted transport of messenger RNA and local protein synthesis near the synapse are important for synaptic plasticity. In order to gain an overview of the composition of the dendritic mRNA pool, we dissected out stratum radiatum (dendritic lamina) from rat hippocampal CA1 region and compared its mRNA content with that of stratum pyramidale (cell body layer) using a set of cDNA microarrays. RNAs that have over-representation in the dendritic fraction were annotated and sorted into function groups.

**Results:**

We have identified 154 dendritic mRNA candidates, which can be arranged into the categories of receptors and channels, signaling molecules, cytoskeleton and adhesion molecules, and factors that are involved in membrane trafficking, in protein synthesis, in posttranslational protein modification, and in protein degradation. Previously known dendritic mRNAs such as MAP2, calmodulin, and G protein gamma subunit were identified from our screening, as were mRNAs that encode proteins known to be important for synaptic plasticity and memory, such as spinophilin, Pumilio, eEF1A, and MHC class I molecules. Furthermore, mRNAs coding for ribosomal proteins were also found in dendrites.

**Conclusion:**

Our results suggest that neurons transport a variety of mRNAs to dendrites, not only those directly involved in modulating synaptic plasticity, but also others that play more common roles in cellular metabolism.

## Background

Neurons are highly polarized cells with extensive processes and thousands of synapses sharing one nuclear genome. The ability to supply specific gene products to a distant synapse is crucial for neuronal functions. Although proteins can be made in the cell body and transported to synapses, local synthesis of proteins from dendritic mRNAs provides an efficient mechanism for delivering synaptic proteins where and when they are needed. It has become evident that mRNAs are transported to the dendrite and translated locally near the synapse following the discovery of synapse-associated polyribosome complexes (SPRCs) [1], and local protein synthesis has been demonstrated following synaptic activation [2-8]. Local protein synthesis is important for long-lasting synaptic plasticity and memory, as locally applied protein synthesis inhibitors block branch-specific long-term facilitation in *Aplysia *neuronal cultures [9], and deletion of the dendritic-targeting element in CaMKIIα mRNA impairs synaptic plasticity and memory consolidation [10]. Given the importance of dendritic RNA transport and local protein synthesis, it is surprising that so little is known about the composition of the dendritic RNA pool. It has been estimated that there could be as many as 400 dendritic RNAs in rat hippocampal neurons [11]. So far a few have been shown to localize in dendrites (reviewed in [12-14]). Most of these mRNAs have been identified sporadically through studies on individual genes of interests. They might not be representative for the whole collection of mRNAs that are transported to dendrites.

Previous attempts to systematically study the collection of transported RNAs have been hindered by lack of bioinformatics tools [15,16]. With improvements in bioinformatics and development of commercial microarrays that covers most of the expressed genome, it is now possible to take an unbiased approach to examine the whole dendritic RNA collection. In order to avoid potential contamination and differential amplification associated with polymerase chain reaction (PCR), as well as possibly altered properties of neurons cultured *in vitro*, it would be ideal if one can isolate a significant amount of dendrites directly from the brain. We took advantage of the structural organization of rodent hippocampus – where cell bodies of pyramidal neurons are aligned and separated from dendritic laminas – and dissected out CA1 stratum radiatum that consists of mostly apical dendrites. To avoid contamination from spillover of abundant messages in cell bodies, mRNAs extracted from dendritic laminas was competed with that of cell body layers on a set of microarrays. Only those with increased representations in the dendritic fraction, compared to the cell body fraction were selected as dendritic mRNA candidates. We show here the identification of 154 dendritic mRNA candidates and in situ hybridization of selected messages. In addition to the previously shown classes of mRNAs encoding receptors, cytoskeleton-interacting proteins, and signaling molecules [12,13], our results revealed new classes of mRNAs that are also transported to dendrites. These mRNAs encode factors that are involved in regulating cell adhesion, protein synthesis and degradation, and membrane trafficking.

## Results

### Identification of dendrite-enriched mRNA candidates by microarray study

Because some messages are expressed at much higher levels than others, direct identification of mRNAs extracted from dendritic laminas would likely be biased towards abundant messages. In addition, the dendritic materials we acquire might be contaminated with small amounts of cell bodies. Therefore, we designed our experiment such that minor contaminations from cell bodies will be competed out using the cell body RNA. Our rationale is that since most mRNAs are restricted to the cell body, an mRNA that is targeted to the dendrite should constitute a higher proportion of the total RNA extracted from dendritic laminas than that from cell body layers. Thus if we co-hybridize equal amount, but distinctively labeled, probes prepared from each sample group to a microarray, we should be able to identify dendrite-enriched RNAs based on the relatively signal strength from each label.

**Figure 1:**
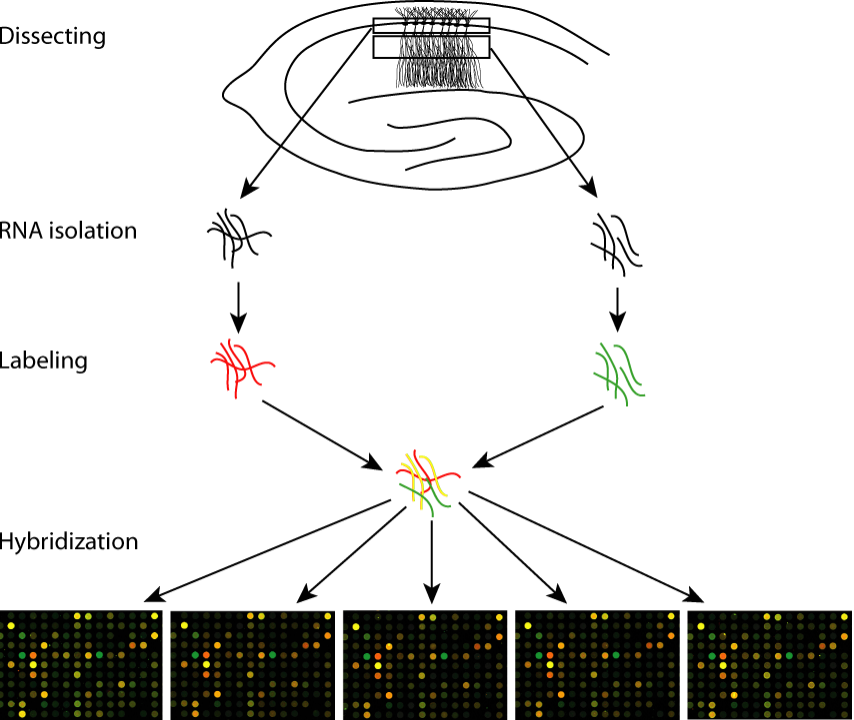
A schematic diagram of the experiment. Stratum pyramidale and stratum radiatum were dissected from hippocampal slices of adult rats. Total RNA extracted from each fraction was reverse primed with a T7 promoter-conjugated oligo-d(T) primer and labeled with either Cyanine 3 or Cyanine 5 through *in vitro *transcription. Equal amount of labeled probes were mixed and hybridized to a set of five replicates of the Agilent 22 K rat oligonucleotide microarray. Enlarged views of the microarrays are presented showing reproducible hybridization.

CA1 stratum radiatum and the corresponding stratum pyramidale of selected hippocampal slices were dissected. A total of one microgram RNA was isolated from dendritic laminas, compared to ten micrograms from the corresponding cell body layers, suggesting that dendrites have a lower concentration of RNA than cell bodies. Five hundred nanograms of each RNA sample was labeled and then hybridized on a set of five replicates of a rat oligonucleotide microarray that each contains more than 22 thousand genes. All five slides showed successful and reproducible hybridization (see Figure 1), and three (Additional files 2, 3, 4) were chosen for further analyses using the Resolver software. Data extracted from these slides were compared to each other. Each replicate showed high levels of consistency with others, with correlation coefficients above 0.95 at a P-value of 0.01. Common features among these data sets were compiled and sorted according to the ratio of dendritic signal versus cell body signal (see Additional file 5). After removing unknown ESTs and entries that are likely due to glial and interneuron contaminations, we generated a list of 154 dendritic RNA candidates that have dendritic signal *vs*. cell body signal ratios equal or greater than 2 (see Additional file 1).

Among these 154 dendritic RNA candidates, 12 encode receptors, ion channels, and postsynaptic proteins (Table 1 and Additional file 1), including a voltage-gated potassium channel, two chlorine channels, and four G protein-coupled receptors (GPCRs). Another 14 messages encode proteins that are involved in regulating cytoskeletons, including a previously known dendritic RNA MAP2 [17] and 12 messages that either directly or indirectly interact with the actin cytoskeleton. Long-lasting modification of synaptic efficacy is usually accompanied by structural changes including outgrowth of existing synapses and formation of new ones [18-20], and such structural changes require modification of the actin cytoskeleton [21,22]. Local synthesis of actin regulators in dendrites could facilitate the control of actin dynamics in response to synaptic activity.

**Table 1:** The dendritic RNA candidates fall in diversified function groups

Category	number	percentage
Receptors, ion channels, and postsynaptic molecules	12	7.8%
Cytoskeleton	12	7.8%
Extracellular matrix, cell adhesion, and immuno-molecules	31	20.1%
Signal transduction and Protein modification	26	16.9%
Translation factors and RNA-binding proteins	7	4.5%
Ribosomal proteins	25	16.2%
Peptide processing and degradation	12	7.8%
Protein transport, membrane trafficking, endocytosis, and exocytosis	10	6.5%
Molecular motor	1	0.6%
Growth factors	4	2.6%
Other	14	9.1%

**Total**	**154**	**100.0%**

Falling into the next category are 29 messages that encode extra-cellular matrix proteins, cell adhesion molecules, and cell surface molecules normally found in immune cells. Adhesion molecules have been best studied in axon path finding and synaptogenesis, however, they may also participate in synaptic plasticity in adults brains, which also involves formation of new synapses and elimination of old ones as mentioned above. It was a little surprising to see immune molecules in our dendritic RNA candidate list. However, it has been reported that the Class I MHC molecules are expressed in mature central nervous system (CNS), and are required for activity-dependent remodeling of synaptic connections [23,24]. Moreover, detection of Class I MHC proteins in synaptosome preparations suggests that they are enriched at synapses [23]. Our results suggest that mRNAs of Class I MHC molecules are transported and locally translated in dendrites. In addition to Class I MHC mRNAs, our list also contains mRNAs for other immune molecules such as the T cell receptor beta chain, which was found in the CNS neurons [25].

### Signaling molecules that are synthesized in dendrites

In the next group we have 27 entries representing 26 mRNAs. These messages are involved in intracellular signaling and protein modification. They include two previously known dendritic mRNAs – calmodulin and G protein gamma subunit [26,27]. There were two different oligos on the microarray representing this G protein gamma subunit mRNA, both had similar ratios of dendrite *vs*. cell body signals (2.31 and 2.19), further suggesting consistency in hybridization and data processing. In addition to G protein and the G protein coupled receptors mentioned above in the receptor category, we also have a few other messages involved in G protein signaling. Several molecules involved in cAMP signaling are also found in this group, including cAMP-dependent protein kinase (PKA), anchor protein for PKA (AKAP), adenylyl cyclase 5, and a PKA substrate that is phosphorylated in response to GPCR activation. Furthermore, other molecules involved in MAPK signaling pathway, ras signaling, CaMK signaling, and PKC signaling are also present. On the phosphatase side, we have two regulatory subunits and one catalytic subunit of the protein phosphatase 1 (PP1). In addition to these signaling molecules, three other messages encode enzymes that are involved in posttranslational protein modification. These enzymes may be important for the maturation of some locally synthesized proteins in dendrites. Finally, we have an mRNA encoding S6 kinase, which could also be appropriately assigned to the next functional group of RNA translational regulators [28].

**Figure 2:**
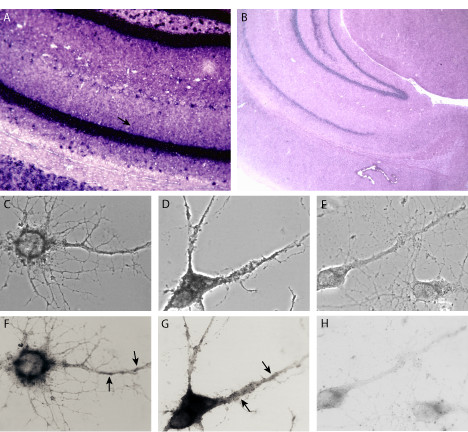
The spinophilin mRNA is transported to dendrites. RNA in situ hybridization on rat brain sections using digoxigen-labeled antisense (A) and sense (B) ribo-probes shows that the spinophilin mRNA is located to the dendritic lamina of hippocampal CA1 region (indicated by an arrow). Phase-contrast (C-E) and plain views (F-H) of in situ hybridization using antisense (C,D,F,G) or sense (F,H) spinophilin probes also revealed its dendritic localization in cultured hippocampal neurons (indicated by arrows). In addition, an increase in spinophilin mRNA level was observed after KCl depolarization (D,G).

Spinophilin is one of the two PP1 regulatory subunits obtained from the screening. It contains an F-actin-binding domain, a PDZ domain, and three coiled-coil structure domains [29,30]. Spinophilin is highly enriched in dendritic spines, and it has been suggested to target PP1 to synapses in addition to regulating PP1 catalytic activity [29]. PP1 is an important protein phosphatase that, among many other functions, regulates AMPA channel activity and synaptic strength, and both activities are modulated by spinophilin [31,32]. In addition to regulating PP1, spinophilin has been suggested to bundle F-actin and link actin filaments to membranes through its actin-binding domain and PDZ domain [30]. Lack of spinophilin leads to increased spine density as well as altered synaptic transmission [33]. Furthermore, spinophilin has also been shown to interact with D2 dopamine receptor [34], adrenergic receptors [35], the trans-Golgi network membrane protein TGN38 [36], and rat lin-10 [37]. It also affects GPCR signaling through its interaction with arrestin [38]. To examine whether the spinophilin mRNA is localized in dendrites, we carried out in situ hybridization using a digoxigen-labeled probe for spinophilin. As shown in figure 2, the spinophilin mRNA is present in dendritic laminas in hippocampus. In addition, the spinophilin mRNA is also localized in the dendrite of cultured hippocampal neurons, and its expression level is elevated after KCl stimulation (Figure 2).

### Messages encoding translation factor eEF1A and translational repressor Pumilio are localized in dendrites

Local translation of dendritic mRNAs is modulated by synaptic activities [2-8]. It is not unexpected to see mRNAs encoding translational regulators are translated locally – and presumably also regulated by synaptic activities – in dendrites. On top of the list for this category is the eukaryotic translation elongation factor 1 alpha (eEF1A) mRNA, which has a dendrite/cell body signal ratio of 4.27. It has been shown that the *Aplysia *eEF1A mRNA can be induced and transported to *Aplysia *sensory neuron axons upon serotonin treatments [16,39]. In addition, the translation of mammalian eEF1A mRNA is regulated by Fragile X mental retardation protein (FMRP) [40], a translational regulator that is required for activity-dependent translation at synapses [6,8]. A recent publication suggests that the mRNA for mammalian eEF1A is indeed localized in dendrites and translated locally upon activation [41].

In addition to eEF1A, mRNAs encoding several RNA binding proteins and a ribonuclease also showed enrichments in the dendritic fraction. These molecules may be involved in RNA metabolism including RNA targeting, translation, and degradation. One of these mRNAs encodes a rat homolog of *Drosophila *Pumilio. Pumilio belongs to a group of maternal factors that determine pattern formation of the *Drosophila *embryo [42]. It acts together with Nanos to repress the translation of Hunchback mRNA [43-46]. In addition, Pumilio has also been suggested to repress Cyclin B1 mRNA in *Xenopus *oocytes [47]. Recently, evidences began to emerge suggesting that Pumilio plays important roles in nerve cells as well. In search for memory-related genes in *Drosophila*, Dubnau *et al*. looked for genes with altered expression levels after learning and genes of which mutation causes memory defects [48]. Pumilio was pulled out from both screening, suggesting it plays an important role in learning. It has been shown that Pumilio binds to the 3' untranslated region (3' UTR) of eIF4E mRNA and regulates its translation at the *Drosophila *larval neuromuscular junction (NMJ) [49]. The level of Pumilio determines postsynaptic eIF4E protein levels, the number and morphology of NMJ boutons, as well as the dendrite morphology of *Drosophila *periphery neurons [49,50]. In addition, Pumilio also modulates neuronal excitability through regulating the mRNA of a voltage-dependent sodium channel [51].

**Figure 3:**
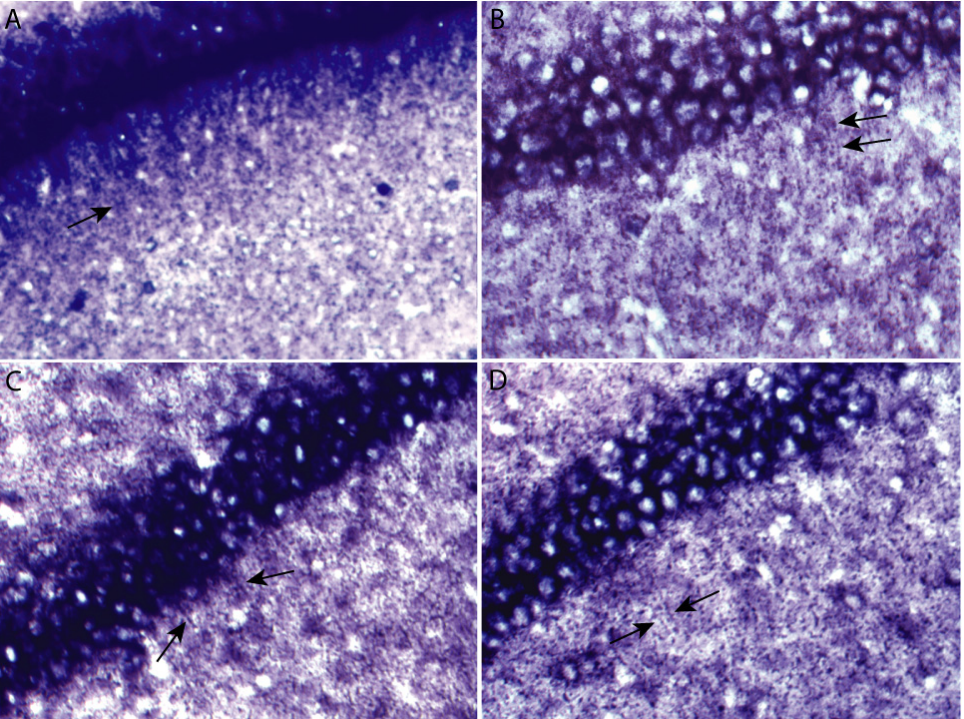
In situ hybridization showing the dendritic localization of the Arc mRNA (A), rat Pumilio 2 mRNA (B), a putative potassium channel I2RF5 mRNA (C), and secretogranin III mRNA (D). Arrows point to punctate staining in the dendritic lamina of rat hippocampal CA1 region.

Our result suggests that Pumilio mRNA is targeted to the dendrite, with a dendrite/cell body signal ratio of 2.15. This is in agreement with its crucial roles in regulating other synaptic messages and in maintaining both synapse morphology and synaptic function. In situ hybridization confirmed that Pumilio mRNA is localized to the dendrite, as manifested by punctate staining in the dendritic lamina of hippocampal CA1 region (Figure 3B). We observed similar punctate staining for several dendritic RNAs, including the Arc mRNA (Figure 3A). These puncta were not due to glial or interneuron staining, as a glia-specific probe and an interneuron-specific probe resulted in very different staining patterns (data not shown). We are not certain whether these puncta are related to the RNA granules reported by other groups [52-56], and we were not able to correlate the size of these puncta with that of RNA granules, as the former were resulted from antibody staining and enzymatic color reactions.

### Peptide processing and protein degradation

A key advantage of protein synthesis in dendrites is that it allows a tighter control of local protein levels at synapses, which cannot be achieved without a counter-balancing mechanism that removes excessive or damaged proteins. The ubiquitin-proteasome has been shown to be important for long-term facilitation in *Aplysia *[57-60], and its components are found in the dendrite of rodent CNS neurons [13,61]. In human, loss-of-function of an E3 ubiquitin ligase E6-AP causes Angelman's syndrome, a disease that is associated with mental retardation [62]. Mice carrying a maternal mutation of this E3 ubiquitin ligase gene exhibit defects in synaptic plasticity and learning [63]. We found that four mRNAs that encode putative E3 ubiquitin ligases and their interacting proteins are enriched in the dendritic fraction, including one responsible for Charcot-Marie-Tooth disease type 1C. Also found in this category are mRNAs of a putative regulatory subunit of the 26S proteasome and several proteases. These molecules could participate in overall protein turnover or targeted degradation of specific proteins, as evidenced by targeted degradation of the regulatory subunit of PKA in *Aplysia *sensory neurons [57]. In addition, ubiquitination could also serve as a signal for endocytosis of membrane proteins like the AMPA receptors, and thus directly modulates the electrophysiological property of synapses [64,65]. Finally, some of these proteases could participate in peptide processing, and may be important for the maturation of some locally synthesized peptides.

### Membrane trafficking and protein transport

Membrane proteins such as adhesion molecules and receptors have to be integrated into synaptic sites, regardless whether they are synthesized in the cell body or in the dendrite. Thus factors involved in membrane trafficking must be present in dendrites. In addition, it is known that levels of some cell surface proteins such as apCAM and AMPA receptors are regulated in activity-dependent manners through endocytic and exocytic pathways [66-68], suggesting that membrane-trafficking factors must be regulated by synaptic activities as well. Our results indicate that mRNAs encoding regulatory factors for membrane trafficking and vesicle transport are present in the dendrite, including several ADP-ribosylation factors. We show here the dendritic localization of secretogranin III mRNA, which encodes a granin protein involved in the secretory pathway (Figure 3D). Furthermore, the mRNA of a myosin regulatory light chain was also isolated from our screening. Myosin is an actin-dependent molecular motor enriched at postsynaptic densities [69]. It may be involved in the transport of RNA granules from the dendritic shaft to dendritic spines [70]. Our result suggests that myosin mRNA is likely targeted to dendrites and translated locally.

### Messages that encode ribosomal proteins are present in dendrites

**Figure 4:**
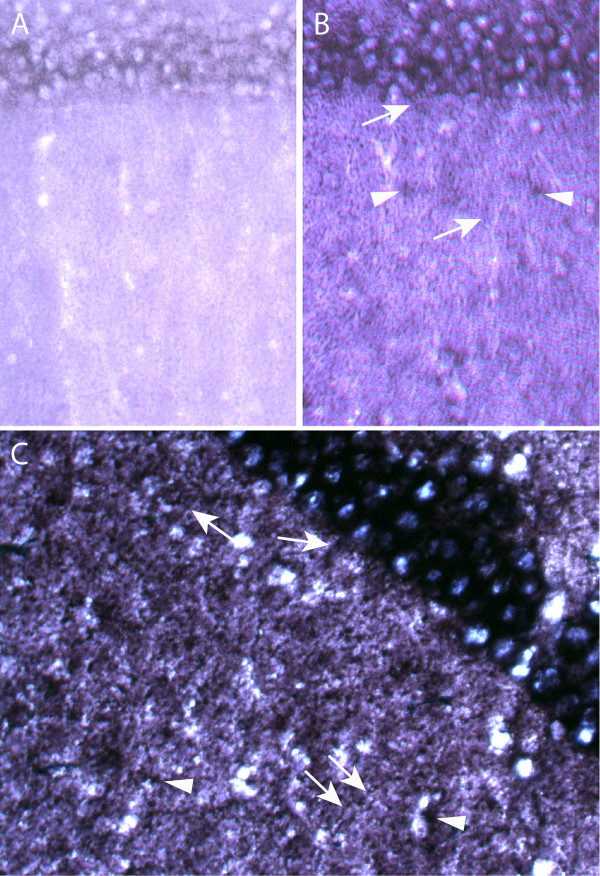
Ribosomal protein L9 mRNA is localized in dendrites and is induced by kainate seizure. In situ hybridization using a sense (A) and an antisense (B,C) probe specific for L9 mRNA, showing L9 mRNA is localized in dendrites of pyramidal neurons (indicated by arrows), in addition to its localization in interneuron and glial cell bodies (indicated by arrowheads). An increased level of L9 mRNA has been observed in brain slices of kainic acid treated animals (C).

In an effort to identify transported mRNAs in *Aplysia *sensory neurons, Moccia and colleagues sequenced a cDNA library constructed from *Aplysia *sensory neuron processes [16]. They showed by in situ hybridization that several ribosomal protein mRNAs are present in *Aplysia *neurites [16]. This is surprising since ribosomal subunits are thought to be assembled in the nucleus [71]. The authors suggested that locally synthesized ribosomal proteins can be added onto partially assembled ribosomal subunits in neurites, and they may be used to replace existing ribosomal proteins in already assembled subunits [16]. In a separate study, Sung et al. also found two mRNAs encoding ribosomal protein S29 and L18a in rat synaptosomes [72]. We have identified 25 ribosomal protein mRNAs in our dendritic RNA list, including L8, S15, S16, and S29 that were identified as transported mRNAs in previous studies [16,72]. To examine whether ribosomal protein mRNAs can be transported to dendrites of mammalian neurons, we carried out in situ hybridization using a probe specific for ribosomal protein L9 mRNA (Figure 4). We observed L9 mRNA signals in dendrites, in addition to the staining of interneuron and glia cell bodies (Figure 4B). Furthermore, we also found that the L9 mRNA can be induced by kainic acid treatment of the animal prior to fixing the brain for in situ hybridization (Figure 4C), suggesting there is an increased demand for L9 protein upon neuronal activation.

## Discussion

We show here the identification of 154 dendritic mRNA candidates in rat hippocampal neurons by comparing the RNA contents of CA1 stratum radiatum and stratum pyramidal. This number does not include 142 unknown ESTs (not shown). In addition, the microarray we were using contains only about two-third of the expressed rat genome. Considering these factors, our number could fall into the same range as the previous estimate of 400 dendritic RNAs by Eberwine and colleagues [11]. These 154 dendritic mRNA candidates represent a broad range of mRNAs that encode diversified functionalities, including receptors and signaling molecules that regulate the electrophysiological properties of the synapse, adhesion molecules and actin cytoskeleton that maintain the structural integrity of the synapse, molecular motors that transport cargos between synapses and cell soma, as well as factors that regulate mRNA translation, protein modification, membrane trafficking, and protein degradation. Clearly, this list does not include all dendritic mRNAs in hippocampal neurons due to technical limitations. Nonetheless, our results provided a much broader view of the dendritic mRNA pool in hippocampal neurons of adult rat brain, and may contribute to our understanding of the *in vivo *biology of these mature neurons.

### Independent mechanisms that target both spinophilin mRNA and protein to dendrites

Our results indicated that the mRNAs of spinophilin, Pumilio, eEF1A, and MHC class I are localized in dendrites. These messages have been shown to play important roles in synaptic plasticity and memory, and their protein products have been found at synapses. We suggest that targeted transport of these messages in dendrites may contribute to the synaptic localization of their protein products.

It has been shown that the actin-binding domain of spinophilin is necessary and sufficient for targeting its protein to dendritic spines [73]. Taken together with our findings, these results suggest that there may be redundant mechanisms of targeting both the mRNA and the protein to ensure sufficient delivery of spinophilin to synapses, a scenario that is analogous to CamKIIα. Deletion of the dendritic-targeting element in CamKIIα mRNA resulted in loss of dendritic localization of the mRNA and a reduction, but not elimination, of the protein at synapses [10], suggesting that CamKIIα protein synthesized in the cell body can find its way to synapses, albeit at a lower level. However, such a decrease in synaptic CamKIIα protein level and/or lack of activity-dependent translation of the CamKIIα mRNA at synapses were sufficient to cause defects in synaptic plasticity and memory consolidation [10], suggesting that these redundant targeting mechanisms are important.

### New classes of messages in dendrites

Previous studies have revealed that mRNAs encoding receptors, cytoskeleton-interacting proteins, and signaling molecules are targeted to the dendrite of mature neurons in rodents [12,13]. In addition, Moccia *et al*. have identified 18 messages in *Aplysia *neurites that fall into the categories of cytoskeleton, translation, transmitter, and adhesion [16]. We find new classes of mRNAs involved in membrane trafficking and protein degradation are also present in dendrites. Our results show that a broad range of proteins can be synthesized in the dendrite, not only those critical for regulating the structural and electrophysiological properties of the synapse, but also others that play more common roles such as protein degradation. We believe that not every dendritic mRNA is tightly regulated by synaptic activity, and some of these messages may be transported because of efficiency rather than necessity, since it may be more efficient to transport mRNAs that each can be used to synthesize multiple copies of the protein.

We were surprised by the finding of ribosomal protein mRNAs in dendrites, even though it is in agreement with earlier findings of these messages in synaptosome preparations and in *Aplysia *neurites [16,72]. We are not certain why these messages are present in dendrites. One possibility is that locally synthesized ribosomal proteins are used for ribosomal protein add-on or replacement in dendrites, as suggested by Moccia *et al*. [16]. Although Moccia *et al*. have emphasized on its potential role in regulating ribosomal activity, we suggest that ribosomal protein replacement could also function in ribosome repair. We know that ribosome assembly is a complex process that involves the addition of a large number of ribosomal proteins to ribosomal RNAs [71], but little is known about what happens if one of these ribosomal proteins gets damaged. Is the whole ribosome then targeted for degradation, or can it be rescued by replacing the damaged protein? The presence of ribosomal protein mRNAs in dendrites suggests that the later possibility could be true.

### Punctate staining and RNA granules

It has been suggested that dendritic RNA may be transported in a very large form of ribonucleoprotein (RNP) complex called RNA granule [52,53]. In addition to the mRNA cargos, RNA granules also contain ribosome, translation factors, RNA-binding proteins, and molecular motors [52-56,74]. Movements of granule-like structures that contain staufen and FMRP have been visualized in the processes of living neurons [54,55,74]. We have observed punctate staining in the dendritic lamina of hippocampus after in situ hybridization using digoxigen-labeled ribo-probes for several mRNAs, including Arc. Punctate staining patterns of the Arc mRNA were also evident following different stimulation paradigms by Steward et al [75]. It is possible that these punctate staining patterns are related to RNA granules. However, in situ hybridization using a BC1-specific probe also showed discontinued (punctate) labeling patterns in the dendrite of cultured neurons [76], even though BC1 RNA did not co-sediment with RNA granules on a sucrose gradient [53]. These results suggest that these puncta could also represent RNA-rich pockets in dendrites that are separated by regions that lack RNA, e.g. membranous structures such as the trans-Golgi network. A possible correlation between the punctate staining patterns and RNA granules has to be further investigated by more detailed co-localization studies of dendritic mRNAs and other components of RNA granules, either in neurons or on a sucrose gradient.

## Conclusion

We have identified 154 dendrite-enriched mRNA candidates in rodent hippocampal neurons. Our results suggest that dendritic mRNAs encode diversified functionalities in neurons.

## Methods

### Dissecting apical dendrites of rat hippocampal CA1 pyramidal neurons

Ten 6–8 week-old male Sprague-Dawley rats (Hilltop Lab Animals, Scottsdale, PA) were euthanized via carbon dioxide inhalation. Hippocampi were dissected out, cut into 300 μm coronal sections, and submerged in *RNAlater *solution (Ambion, Inc., Austin, TX) in order to stabilize RNA during dissection. Cell nuclei were stained with 5 μM SYTO 24 (Molecular Probes, Inc., Eugene, OR) to guide the dissection and to avoid contamination from cell bodies. CA1 stratum radiatum and the corresponding stratum pyramidale were dissected under a dissecting microscope with a UV light source. Only slices with tight organization of stratum pyramidale and little nuclear staining in stratum radiatum were used. Cuts were made above the lacunosum-moleculare layer to avoid scattered cell bodies from this region.

### Probe preparation and microarray analysis

Dissected stratum radiatum and stratum pyramidale from 10 individual rats were pulled for RNA extraction using an RNeasy Protect Mini Kit from Qiagen (Valencia, CA). The integrity of RNA samples was examined using a Bioanalyzer (Agilent Technologies, Palo Alto, CA). Five hundred nanogram of each RNA sample was reverse-transcribed using a T7 promoter-conjugated oligo-dT primer, linear-amplified and labeled with either Cyanine 3 – CTP or Cyanine 5 – CTP using a Low RNA Input Fluorescent Linear Amplification Kit (Agilent Technologies, Palo Alto, CA). Equal amounts of Cy3 and Cy5-labeled cRNA probes were mixed, and hybridized to a set of five replicas of a 22 K rat oligonucleotide microarray (Agilent Technologies, Palo Alto, CA) following the instructions supplied by the manufacturer. The resulting slides were scanned using a microarray scanner. Feature extraction, including standardization of signal intensity, background correction, and statistical analyses, were performed using Agilent's Feature Extraction Image Analysis Software following protocols from the manufacturer. Data from each individual array were compared and further analyzed using the error model of the Resolver software (Rosetta, Inc., Seattle, WA) to generate the final result. Messages that have at least 2-fold enrichment in the dendritic fraction over the cell body fraction were annotated using the GO_function and YPD_function databases in addition to the Agilent annotation. Each entry was then compared with published literatures and sorted into various groups based on its potential function. Entries that are likely resulted from glial and interneuron contaminations were removed from the list.

### RNA in situ hybridization

Gene accession numbers were used to download the cDNA sequences from Genebank. Primers were designed to amplify 250–450 bp cDNA fragments from rat brain RNA by RT-PCR. The resulting PCR fragments were cloned into the pCRII-TOPO vector using a TOPO TA cloning kit (Invitrogen, Carlsbad, CA). The identity of each clone was confirmed by sequencing, and plasmid DNA was used to generate digoxigen-labeled sense and antisense ribo-probes through *in vitro *run-off transcription. A spinophilin cDNA clone was received as a gift from Dr. Patrick Allen, and was used to generate spinophilin-specific probes. Four to six week-old male Sprague-Dawley rats (Hilltop Lab Animals, Scottsdale, PA) were treated with or without subcutaneous injection of 25 mg kainic acid (Sigma-Aldrich, St. Louis, MO) per kg of body weight. Fresh-frozen brains were cut into 20 μm coronal sections and hybridized according to the published protocol with modifications [77].

## List of abbreviations

MAP2: Microtubule associated protein 2

EEF1A: eukaryotic translation elongation factor 1 alpha

CaMKIIα: calcium/calmodulin-dependent protein kinase (CaM kinase) II alpha

EST: expressed sequence tag

GPCR: G protein-coupled receptor

PKA: cAMP-dependent protein kinase

## Authors' contributions

JZ carried out experimental design, dissection, sample preparation, and microarray hybridization (with the help of LMB). JZ also carried out in situ hybridization, literature search, and manuscript preparation. TZ carried out gene annotation and functional database search. LMB helped with microarray hybridization, and carried out microarray scanning, data processing, and statistical analyses. This study was conceived by JZ.

## Supplementary Material

Additional File 1 An Excel file containing the complete list of 154 dendritic mRNA candidates. Each sequence was annotated with three databases, and subjected to literature search. Relevant PubMed IDs were also included. Click here for file

Additional File 2 Original results from three microarrays, including gene ID, log ratio, log ratio error, P value, and signal intensities, signal error, mean signal, median signal, and pixel S Dev for both red and green signals. Click here for file

Additional File 3 Original results from three microarrays, including gene ID, log ratio, log ratio error, P value, and signal intensities, signal error, mean signal, median signal, and pixel S Dev for both red and green signals. Click here for file

Additional File 4 Original results from three microarrays, including gene ID, log ratio, log ratio error, P value, and signal intensities, signal error, mean signal, median signal, and pixel S Dev for both red and green signals. Click here for file

Additional File 5 Final processed data for all 22 K entries, including signal intensities, log ratio, log ratio error, and fold change. Click here for file
